# Role of Primary Autologous Bone Graft at Docking Site in the Treatment of Infected Non-union Tibia Using Rail Fixation System

**DOI:** 10.5704/MOJ.2103.005

**Published:** 2021-03

**Authors:** S Mudiganty, J Austine

**Affiliations:** 1 Department of Trauma and Orthopaedics, East Lancashire Hospitals NHS Trust, Blackburn, United Kingdom; 2 Department of Orthopedic Surgery, Jawaharlal Institute of Postgraduate Medical Education and Research (JIPMER), Puducherry, India

**Keywords:** non-union, rail fixation, acute docking, bone grafting

## Abstract

**Introduction:**

Distraction osteogenesis has been used effectively in the management of tibia non-unions with skeletal defect. A retrospective case series study of the infected non-union tibia managed with acute docking in a rail fixation system was conducted at a tertiary care hospital in South India. It was designed to evaluate the use of autologous bone graft at the docking site in achieving an early union with a seven years follow-up period.

**Materials and Methods:**

From 2010 to 2017, a total of 19 patients with infected tibia non-union and a bone defect less than 3cm, were treated with debridement and a monolateral frame fixation with acute shortening and lengthening. The patients were divided into two groups: one in which no bone graft was used at the docking site during early years of the study; and a later group in which autologous bone graft was used at the acute docking site primarily in addition to compression. Consolidation at the docking site was assessed both radiographically and clinically, and the results were statistically analysed.

**Results:**

There were 12 patients in Group I without bone graft, where consolidation at the docking site was noted after a mean duration of 22.08 ± 3.87 weeks. There were seven patients in Group II with bone graft, where the mean time for docking site consolidation was significantly lower at 16.57 ± 3.82 weeks. No docking site complications were noted in either group.

**Conclusion:**

Primary autologous bone graft enhances docking site consolidation in acute shortening. The routine use of bone graft at the docking site in acute shortening will expedite the docking site union with reduction of treatment time.

## Introduction

The management of non-unions of the tibia with a skeletal defect is a big challenge for the orthopaedic surgeons. Distraction osteogenesis has been used effectively to resolve this problem. This technique involves a corticotomy above or below the defect followed by progressive transport of the normal bone segment into the bone defect. The gap created at the corticotomy site is filled with new bone called regenerate. The junction of the healthy bone segment and the end segment is called the docking site. Defects of the tibia measuring less than 3cms are treated with acute docking and lengthening wherein the bone defect is acutely closed and length achieved by distraction at the corticotomy site^1-4^.

Union at the docking site is a major hindrance for early fixator removal and successful completion of treatment^5,6^. An optimal technique of achieving 100% docking site union has yet to be described. The opposition of bone ends in acute docking could achieve early docking site union, with a reduction in treatment time^7^. With a view of further enhancing the union at the docking site in acute shortening, and thereby curtailing treatment time, the routine use of bone graft primarily at the docking site has been proposed by Tetsworth *et al*^8^.

The fundamental objective of this study is to evaluate the use of autologous bone graft at the docking site in achieving an early union. Two groups of patients both treated for infected tibia gap non-unions with acute docking and lengthening, were retrospectively evaluated. One group received autologous bone graft at the docking site primarily and the other group was treated with compression alone. We hypothesised that the use of autologous bone graft enhanced consolidation at the docking site.

## Material and Method

This retrospective case series study was approved by the institutional ethics committee. From June 2010 to July 2017 a total of 19 patients with infected tibia non-union who were treated with debridement and monolateral frame fixation were enrolled into the study. The inclusion criteria were all skeletally mature adults, with bone defect of less than 3cms and who were treated with acute shortening and lengthening. Patients with bone defects more than 3cms and/or soft tissue defects that would lead to bone exposure even after the shortening were excluded from the study. Out of the 19 patients, 13 were male and 6 were female with ages varying from 18 to 56 years (mean 36.3 years). Thirteen cases involved the right leg and in six patients the left leg was affected. The infected non-union was a sequel to an open fracture in twelve patients; closed fracture treated with intramedullary nail fixation with subsequent infection in four patients; and chronic osteomyelitis in three patients. In all 19 cases the defect was located in the diaphysis of the distal tibia and the corticotomy was performed in the proximal tibia metaphysis region. If the fibula was intact (nine cases), then a fibulectomy was performed to aid in acute shortening.

The patients were followed up every four weeks with a standard anteroposterior and lateral radiograph of the affected leg for the duration of treatment. During the early years of the study, from 2010 to 2013, no bone graft was used at the docking site. Union at the docking site was achieved through compression alone. In the later half of the study, autologous bone graft was used at the acute docking site primarily, in addition to compression, in all patients except those who refused to consent for the bone graft procedure. The 12 patients who refused bone grafting, were placed in Group I, and those who consented and received a bone graft, 7 patients , were clustered into Group II.

In Group I, the number of males was eight and females was seven. Seven patients had bone defect secondary to an open fracture, three patients had chronic osteomyelitis and two patients had a post-operative infection leading to a bone defect. Group II had five males and two females. Bone defect as a sequel to an open fracture was noted in five of these patients, and for two patients, each had chronic osteomyelitis and bone defect due to a post-operative infection.

The surgical technique employed was the same in both the groups. All patients underwent a meticulous debridement and acute docking with or without bone grafting. All procedures were carried out as a single stage. The antibiotic protocol was the same in both the groups. Fish scaling was done in all cases at the docking site. Autologous cancellous bone graft was obtained from the iliac crest. Radiographic union at the docking site was completed when 3 out of 4 cortices showed consolidation on the two routine views obtained. Once radiographic union was confirmed, clinically union was tested by docking site manual stress. The monolateral frame was removed once clinical and radiographic union was achieved at the docking site and the regenerate.

## Results

This retrospective case series study was approved by the institutional ethics committee. From June 2010 to July 2017 a total of 19 patients with infected tibia non-union who were treated with debridement and monolateral frame fixation were enrolled into the study. The inclusion criteria were all skeletally mature adults, with bone defect of less than 3cms and who were treated with acute shortening and lengthening. Patients with bone defects more than 3cms and/or soft tissue defects that would lead to bone exposure even after the shortening were excluded from the study. Out of the 19 patients, 13 were male and 6 were female with ages varying from 18 to 56 years (mean 36.3 years). Thirteen cases involved the right leg and in six patients the left leg was affected. The infected non-union was a sequel to an open fracture in twelve patients; closed fracture treated with intramedullary nail fixation with subsequent infection in four patients; and chronic osteomyelitis in three patients. In all 19 cases the defect was located in the diaphysis of the distal tibia and the corticotomy was performed in the proximal tibia metaphysis region. If the fibula was intact (nine cases), then a fibulectomy was performed to aid in acute shortening.

The patients were followed up every four weeks with a standard anteroposterior and lateral radiograph of the affected leg for the duration of treatment. During the early years of the study, from 2010 to 2013, no bone graft was used at the docking site. Union at the docking site was achieved through compression alone. In the later half of the study, autologous bone graft was used at the acute docking site primarily, in addition to compression, in all patients except those who refused to consent for the bone graft procedure. The 12 patients who refused bone grafting, were placed in Group I, and those who consented and received a bone graft, 7 patients , were clustered into Group II.

In Group I, the number of males was eight and females was seven. Seven patients had bone defect secondary to an open fracture, three patients had chronic osteomyelitis and two patients had a post-operative infection leading to a bone defect. Group II had five males and two females. Bone defect as a sequel to an open fracture was noted in five of these patients, and for two patients, each had chronic osteomyelitis and bone defect due to a post-operative infection.

The surgical technique employed was the same in both the groups. All patients underwent a meticulous debridement and acute docking with or without bone grafting. All procedures were carried out as a single stage. The antibiotic protocol was the same in both the groups. Fish scaling was done in all cases at the docking site. Autologous cancellous bone graft was obtained from the iliac crest. Radiographic union at the docking site was completed when 3 out of 4 cortices showed consolidation on the two routine views obtained. Once radiographic union was confirmed, clinically union was tested by docking site manual stress. The monolateral frame was removed once clinical and radiographic union was achieved at the docking site and the regenerate.

**Table I:** Data showing mean, standard deviation and p value

	Time (weeks)			
Group	N	Median	Interquartile range	
Without bone graft	12	23	7	
With bone graft	7	16	6	p < 0.05

**Fig. 1:**
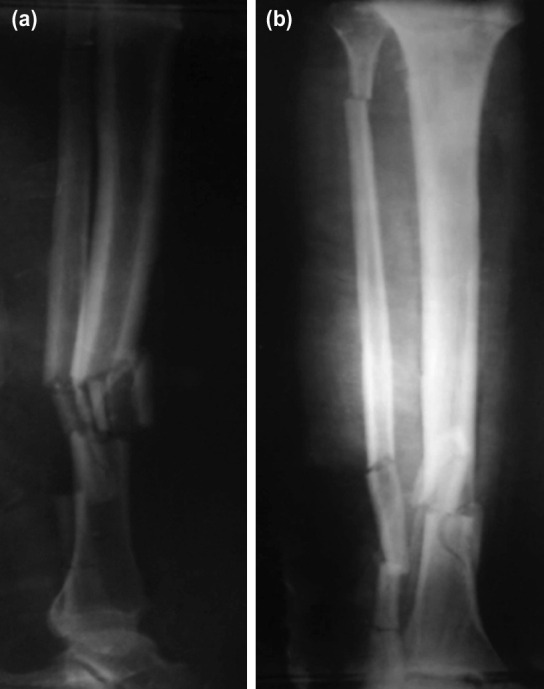
(a) Lateral and (b) anterior posterior plain radiograph of open fracture of the tibia.

**Fig. 2:**
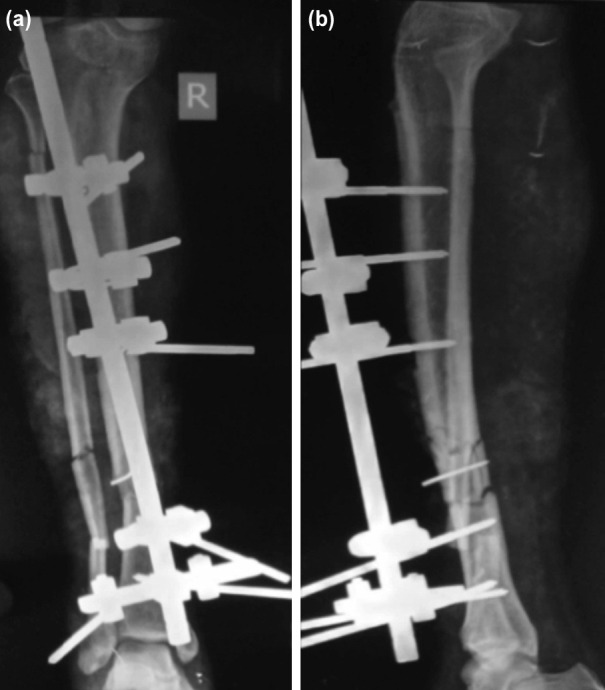
(a) Anterior posterior and (b) lateral plain radiograph showing tibia fracture treated with debridement and external fixation.

**Fig. 3:**
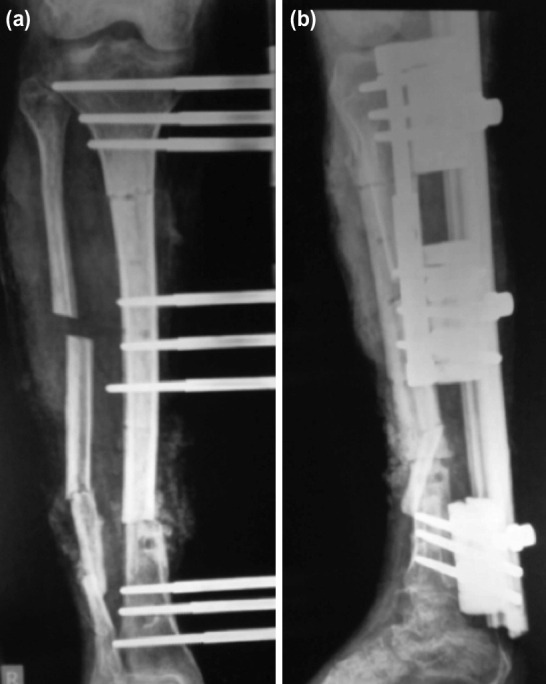
(a) Anterior posterior and (b) lateral plain radiograph showing tibia fracture treated with Debridement, acute docking, primary bone graft and proximal corticotomy.

**Fig. 4:**
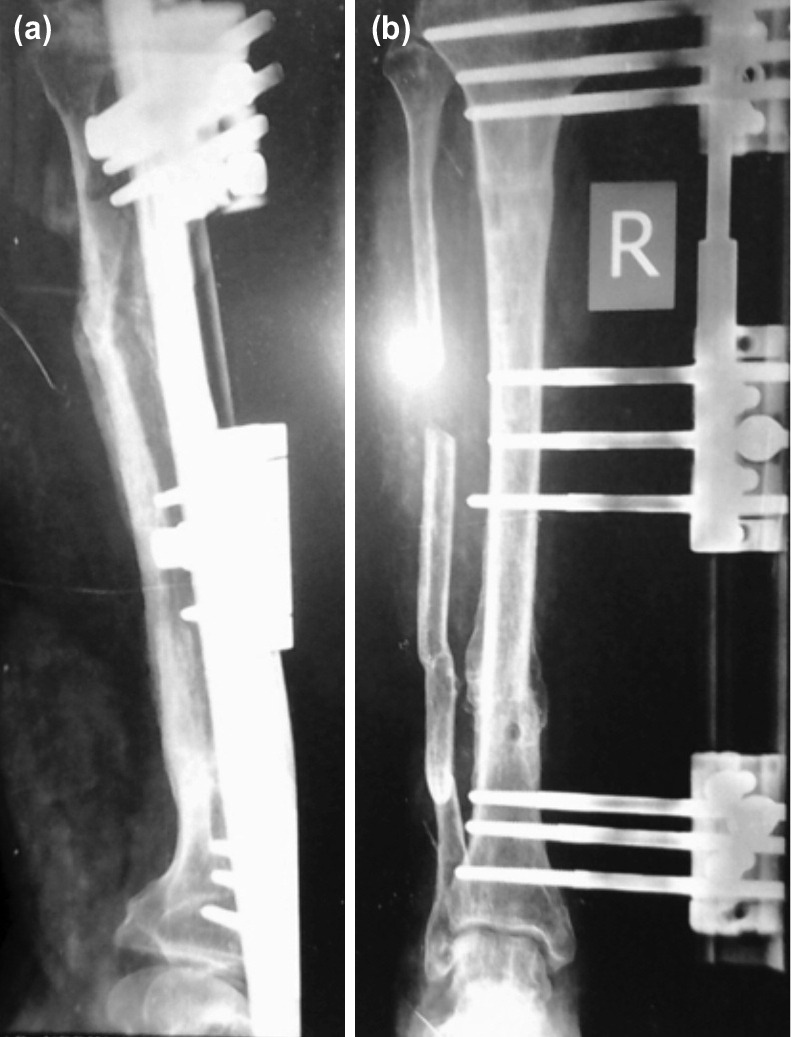
(a) Anterior posterior and (b) lateral plain radiograph showing Union achieved.

## Discussion

It is a well-known fact that when bone loss is less than 3cms in the tibia, then the treatment of choice is an acute shortening and lengthening^1-4^. Acute docking covers the bone gap immediately and distraction at the corticotomy site achieves the necessary length. This technique has numerous advantages compared to bone transport, namely excellent union without the need for additional procedures^9,10^, fewer complications^4,8^, strong mechanical construct^4^, maintainence of alignment^11^, superior radiological results^8^, and enhanced patient gratification due to reduced external fixator time^4,10^.

It has been proposed that the healing of the docking site differs from that of the regenerate^12^. The regenerate heals by intramembranous ossification, and in contrast, the docking site takes a longer time for union with endochondral ossification and creeping substitution. Therefore, the docking site and the regenerate are two distinctive biological phenomena. Healing of the docking site is a major concern and is responsible for prolonging the external fixator time^5,6^. Thus, union of the docking site can be considered as a rate limiting step in the entire procedure1,6. Bone grafting at the docking site in cases of bone transport is advocated by a number of authors^6,10,13^. In cases of acute shortening and lengthening, Mahaluxmivala *et al* report a 100% union at the docking site with no additional procedure^10^. In contrast, Tetsworth *et al*^8^ observed a 38% incidence of delayed union or non-union at the docking site in the acute shortening cases. They recommend the routine use of bone graft at the docking site in acute shortening cases as this expedites the docking site union potentially reducing treatment time.

Autologous bone graft is the accepted method of enhancing union of the regenerate and the docking site^7,14^. It is well known that only a bone graft has osteoinductive, osteogenic and osteoconductive qualities^15^. However, the negative point of autologous bone graft is the need for an additional procedure and accompanying donor site morbidity^16^. Hatzokos et al report similar rates of docking site union in bone transport cases with the use of demineralised bone matrix and autologous bone marrow in comparison to autologous bone graft thereby minimising donor site morbidity^17^.

In our study, we aimed to highlight the routine use of primary autologous bone grafting at the docking site in cases of acute shortening and lengthening with improved docking site consolidation. Our results prove that union at the docking site was much faster with the use of autologous bone graft in comparison to compression alone. In all our cases the docking site was located in the diaphysis eliminating the bias of docking site region. Moreover the use of monolateral frames in all our cases removes the bias associated with frame design. Our results are consistent with the outstanding consolidation results reported with the use of autologous bone graft in management of tibial non-unions^18^.

The retrospective design of this study is one of its limitations along with the fact that the two treatment strategies were employed sequentially rather than randomly. Surgical expertise gained over a period of time could have positively influenced the results of the bone grafting group. Dahl et al pointed out the influence of surgical expertise on distraction osteogenesis cases^19^. Initially the docking site was treated with compression alone and primary autologous bone grafting was employed later on. Our study focused on the docking site union with the assumption that if it heals faster, then the external fixator index will be shortened. However, the external fixator index was not calculated or compared. Further studies are needed to evaluate the advantage of docking site bone graft on the functional outcome at the end of the treatment.

## Conclusion

The use of autologous bone graft primarily at the docking site in cases of acute shortening hastens docking site consolidation in comparison to docking site treatment with compression alone.
